# Type 2 diabetes microenvironment promotes the development of Parkinson’s disease by activating microglial cell inflammation

**DOI:** 10.3389/fcell.2024.1422746

**Published:** 2024-07-10

**Authors:** Bohan Zhang, Chengyuan Song, Xiao Tang, Min Tian, Yuqian Liu, Zhuoran Yan, Ruonan Duan, Yiming Liu

**Affiliations:** Department of Neurology, Qilu Hospital, Shandong University, Jinan, China

**Keywords:** Parkinson’s disease, type 2 diabetes, inflammation, mitochondria, cGAS-STING

## Abstract

**Objective:**

Parkinson’s disease (PD) is the second most common neurodegenerative disease in the world, and type 2 diabetes (T2DM) and PD are influenced by common genetic and environmental factors. Mitochondrial dysfunction and inflammation are common pathogenic mechanisms of both diseases. However, the close association between PD and T2DM and the specific relationship between them are not yet clear. This study aimed to reveal the specific connection between the two diseases by establishing a mouse model of comorbid PD and T2DM, as well as a Bv2 cell model.

**Methods:**

C57BL/6 mouse were used to construct a model of PD with T2DM using streptozotocin and rotenone, while Bv2 cells were used to simulate the microenvironment of PD and T2DM using rotenone and palmitate. Behavioral tests were conducted to assess any differences in motor and cognitive functions in mouse. Immunohistochemistry was used to analyze the number of dopaminergic neurons in the substantia nigra region of mouse. Western blotting was used to detect the expression levels of TH, P-NFκB, NFκB, Cyclic GMP-AMP synthase (cGAS), and Stimulator of interferon genes (STING) proteins in the substantia nigra region of mouse and Bv2 cells. qRT-PCR was used to analyze the expression levels of IL1β, IL6, and TNF-α. Seahorse technology was used to assess mitochondrial function in Bv2 cells.

**Results:**

T2DM exacerbated the motor and cognitive symptoms in mouse with PD. This effect may be mediated by disrupting mitochondrial function in microglial cells, leading to damaged mtDNA leakage into the cytoplasm, subsequently activating the cGAS-STING pathway and downstream P-NFκB/NFκB proteins, triggering an inflammatory response in microglial cells. Microglial cells release inflammatory factors such as IL1β, IL6, and TNF-α, exacerbating neuronal damage caused by PD.

**Conclusion:**

Our study results suggest that T2DM may exacerbate the progression of PD by damaging mitochondrial function, and activating microglial cell inflammation. The detrimental effects on Parkinson’s disease may be achieved through the activating of the cGAS-STING protein pathway.

## 1 Introduction

Parkinson’s disease (PD) is a common neurodegenerative disease in middle-aged and elderly individuals, characterized by bradykinesia, resting tremor, and rigidity as the main clinical features (Dorsey et al., 2018a). The major pathological changes involve the gradual degeneration and damage of dopaminergic neurons in the substantia nigra, locus coeruleus, and striatum (Deuschl et al., 2020). As of 2016, approximately 61 million people worldwide had Parkinson’s disease, imposing a heavy burden on caregivers and society (Dorsey et al., 2018b).

Type 2 diabetes mellitus (T2DM) is characterized by impaired pancreatic β-cell function and insulin resistance (Patel et al., 2024). The development of both PD and T2DM is influenced by common environmental and genetic factors, and both are associated with mitochondrial dysfunction and inflammation (Lima et al., 2022). Furthermore, numerous clinical studies have shown that T2DM can increase the risk of cognitive impairment and dementia in patients, although the specific mechanisms remain unclear (Luo et al., 2022). Patients with T2DM often experience a high-sugar, high-fat, and chronically inflammatory environment, and research has suggested that T2DM can exacerbate the occurrence and progression of neurodegenerative diseases by inducing neuroinflammation (Verdile et al., 2015; Chen et al., 2016). Mitochondrial damage and neuroinflammation are also significant factors in the development of Parkinson’s disease. One research has shown that mitochondrial damage leads to morphological changes, leakage of mitochondrial DNA, inadequate energy supply to neurons, and exacerbation of neuronal damage (Hakiminia et al., 2022).

In patients with Parkinson’s disease, *postmortem* examinations have revealed activated microglial cells in the substantia nigra region, elevated levels of inflammatory factors, and evidence of microglial cell activation and neuronal loss in imaging studies (Marinova‐Mutafchieva et al., 2009). The release of numerous inflammatory factors from microglial cells further worsens neuronal damage, promoting the occurrence and progression of Parkinson’s disease (Tansey and Goldberg, 2010). The cGAS-STING pathway consists of three main proteins: cGAS, cGAMP, and STING. Cyclic GMP-AMP synthase (cGAS) is a double-stranded DNA sensor composed of a nucleotidyl transferase domain and two major DNA-binding domains (Wu et al., 2014). In situations where the cell nucleus or mitochondria are damaged, nuclear DNA and/or mitochondrial DNA (mtDNA) enter the cytoplasm, leading to the binding of cGAS to DNA, catalyzing the synthesis of cyclic GMP-AMP (cGAMP) from adenosine triphosphate (ATP) and guanosine triphosphate (GTP) (Shu et al., 2014). The synthesized cGAMP induces a conformational change in Stimulator of interferon genes (STING), activating it. Activated STING is then transported from the endoplasmic reticulum (ER) to the Golgi apparatus, where it activates transcription factor nuclear factor-kB(NFκB), leading to the expression of interferons and inflammatory cytokines such as Tumor necrosis factor-α(TNFα), Interleukin-1β(IL-1β), and Interleukin-6(IL-6) (Kato et al., 2017; Ritchie et al., 2022). In aging-related research, the cGAS-STING pathway has been closely linked to aging and neurodegenerative diseases (Hamann et al., 2019; Yu et al., 2022). Our previous studies have shown that patients with PD and T2DM experience more severe cognitive impairments, highlighting the need for further research into the mechanisms by which T2DM impacts the occurrence and progression of PD.

Bv2 cell line, derived from C57BL/6 mouse, is a commonly used microglial cell line in neuroinflammation research, while SH-SY5Y cells, derived from neuroblastoma, are widely used in studies related to neurodegenerative diseases (Sarkar et al., 2018; Hoffmann et al., 2023). C57B6L mouse are the most frequently used mouse research models. Streptozotocin (STZ) is commonly used to construct mouse models of T2DM, while palmitate acid (PA) is used to simulate the T2DM environment *in vitro* cell models. Rotenone is a commonly used drug for constructing PD models (Sun et al., 2018). In this study, we utilized STZ and rotenone to construct a model of PD with T2DM, investigating the phenotypic and dopaminergic neuronal effects of T2DM on PD mouse through behavioral experiments. Additionally, by combining Bv2 cells to create a cell model in a T2DM environment with PD, we explored the effects of T2DM on mitochondrial function and inflammation in PD, determining the specific relationship between the two. Therefore, this study elucidates the pathophysiological connection between PD and T2DM, confirming that T2DM can exacerbate PD.

## 2 Materials and methods

### 2.1 Construction of Parkinson’s disease mouse model with type 2 diabetes

Four-week-old C57BL/6J male mouse (Beijing Vital River Laboratory Animal Technology Co., Ltd.) were selected as experimental animals and divided into four groups: WT group, T2DM group, PD group, and PD with T2DM group. The WT group and PD group were fed normal mouse chow for 8 weeks (Beijing Vital River Laboratory Animal Technology Co., Ltd.) followed by injection of citrate buffer at a dose of 0.1 mL per 10 g body weight. The T2DM group and PD with T2DM group were fed a high-fat diet (Beijing Vital River Laboratory Animal Technology Co., Ltd.) for 8 weeks, followed by two consecutive days of intraperitoneal injection of a STZ solution with a concentration of 1 mmol/mL at a dose of 0.1 mL per 10 g body weight. Blood glucose levels were measured 7 days later, and blood glucose levels exceeding 13.9 mmol/L were considered successful in establishing the T2DM model (Winzell and Ahrén, 2004). Subsequently, the PD group and PD with T2DM group were orally gavaged with a solution of rotenone with a concentration of 0.5 mg/mL for 8 weeks, while the WT group and T2DM group were orally gavaged with 0.5% carboxymethyl cellulose sodium for 8 weeks, both at a dose of 0.1 mL per 10 g body weight.

### 2.2 Rotarod test

The rotarod test is commonly used to assess the motor abilities of mouse (Shiotsuki et al., 2010). For 3 days prior to the experiment, mice are trained by placing them on a rotating rod set at an initial speed of 4 rpm for 1 min. The speed is then increased to 10 rpm for 2 min, followed by further acceleration to 30 rpm for an additional 2 min. During the actual experiment, the initial speed of the rotating rod is set at 4 rpm with acceleration rate of 20 rpm/min until reaching a maximum speed of 40 rpm. The maximum duration is 5 min, and the time taken for the mouse to fall off the rod is recorded. If the mouse has not fallen off by the end of 5 min, it is placed back in its cage. Each mouse is tested three times, and the average time is calculated.

### 2.3 Balance beam test

The balance beam test is widely used to assess the limb coordination and motor impairment of mouse (Luong et al., 2011). In this test, mouse are required to crawl from one end of a 3 cm wide, 50 cm long beam to the other end. Prior to the formal experiment, mice are trained for 3 days by placing them at one end of the balance beam, where they typically crawl forward and run uninterruptedly to the other end. The time taken by the mouse to run from one end to the other is recorded. During the formal experiment, mice are placed at the starting end, and the time taken by them to naturally crawl to the other end is recorded. Each mouse repeats the test three times, and the average time is calculated.

### 2.4 Grip strength test

The grip strength test is commonly used to evaluate the forelimb grip strength of mouse using a grip strength meter (Munier et al., 2022). During the test, the mouse’s forepaws are placed on the contact end of the grip strength meter, and the mouse is gently pulled horizontally until it releases the grip strength meter. This process is repeated three times for each mouse, and the average grip strength is calculated.

### 2.5 Barnes maze test

The Barnes maze test is used to study the long-term memory capabilities of mouse (Rodríguez et al., 2024). In the week leading up to the experiment, mice are trained by placing them under a target hole for 2 min to allow them to acclimate. The mouse is then placed in the center of the maze with a non-transparent box restricting movement for 5 s. The box is removed, and the time taken by the mouse to enter the target location is recorded. Each mouse has a maximum training time of 5 min. If the mouse fails to find the target location within the time limit, it is gently placed in the target location for 1 min. Each mouse undergoes continuous training for 5–6 days. During the final experiment, the mouse is placed in the center of the maze within a non-transparent box and allowed to explore. The evasion time is recorded, with a maximum observation time of 5 min. Each animal repeats the test three times, and the average time is calculated.

### 2.6 Immunohistochemistry

The mouse brain tissues were fixed in 4% paraformaldehyde, dehydrated in graded alcohol, xylene, and embedded in paraffin to create paraffin-embedded tissue blocks (Hussaini et al., 2024). The blocks were sectioned at a thickness of 4 mm using a microtome and the sections were collected. The paraffin sections were deparaffinized in xylene and graded alcohol, placed in citrate buffer solution, subjected to high-temperature and high-pressure dewaxing, washed three times with PBS, blocked with goat serum for 30 min, and then incubated with anti-TH (1:1,000,ab112,Abcam) primary antibody at 4°C in the refrigerator for 12 h. After 12 h, the sections were brought to room temperature, washed three times with PBS, incubated with the reaction enhancer poly helper for 30 min, washed three times with PBS, incubated with the secondary antibody for 30 min, subjected to DAB staining, observed under a microscope, and finally sealed with neutral resin.

### 2.7 Cell culture and reagent treatment

The Bv2 and SH-SY5Y cell line, which was produced by the American Type Culture Collection (ATCC, Manassas, VA, United States), was cultured in Dulbecco’s modified Eagle’s high glucose medium (DMEM, 11,965, Life Technologies, Rockville, MD, United States), supplemented with 10% fetal bovine serum (10,099, Invitrogen, CA, United States) in a humidified incubator with 5% CO2 at 37°C. We prepared rotenone (R8875, Sigma-Aldrich, St Louis, MO, United States) in dimethyl sulfoxide (DMSO; D2650, Sigma-Aldrich) at a stock concentration of 50 mM (Dodiya et al., 2020). Palmitate acid (P0169, Sigma-Aldrich, St Louis, MO, United States) was diluted in Citrate solution at a stock of 500 mM. Two chemicals were then diluted with DMEM to a concentration of 1 mM and incubated with cells for different amounts of time (0, 12, 24, 36, and 48 h) according to the corresponding experiments. The normal control (NC) was used as the control.

### 2.8 West blot

Levels of protein expression were measured using western blotting analysis, which was performed using standard methods with commercially available antibodies (Pillai-Kastoori et al., 2020). After grinding mouse brain tissue in the substantia nigra region into a protein homogenate and treating cells, the whole-cell lysate was prepared using lysis buffer, protease inhibitors, and phenylmethylsulphonyl fluoride. The protein concentration was measured using a Bicinchoninic Acid protein assay kit (23,227, Thermo Scientific, Waltham, MA, United States). Soluble proteins (30 mg) were separated using 8%–12% SDS polyacrylamide gels and then transferred onto polyvinylidene difluoride membranes (200 mA, 60 min), which were then blocked in 5% non-fat milk or bovine serum albumin in tris-buffered saline with tween 20 and incubated with primary antibodies overnight. The primary antibodies used were as follows: anti-GAPDH (1:5,000,ab8245,Abcam); anti-βactin (1:5,000,ab8226,Abcam); anti-TH (1:1,000,ab112,Abcam); anti-cGAS (1:1,000,ab302617,Abcam); anti-Sting (1:1,000,ab239074,Abcam); anti-P-NFκB (1:1,000,ab32536,Abcam); anti-NF-κB (1:1,000,ab16502,Abcam). Then, secondary HRP-linked antibodies were used to detect primary antibodies. The immunoreactive signal was detected using an ECL substrate kit (Millipore, Billerica, MA, United States). Gray values of the protein bands were analyzed using ImageJ 1.8.0r software (National Institutes of Health, Bethesda, MD, United States).

### 2.9 RNA isolation and quantitative real-time polymerase chain reaction

RNA was isolated from cells following the manufacturer’s protocol using TRIzol (Invitrogen, 15,596–026), and reverse transcription was used to convert 1 mg RNA to cDNA using a cDNA Synthesis kit (Vazyme, R323-01). Quantitative real-time polymerase chain reaction (qRT-PCR) analysis was performed using the Super Real PreMix Plus SYBR Green (Vazyme, Q711-02) (Bridge, 2017). The relative mRNA expression levels were normalized to those of GAPDH and calculated using the 2−11CT method. The primer sequences for qRT-PCR were as follows: GAPDH: forward:5′-GCACCGTCAAGGCTGAGAAC-3′ and reverse: 5′-TGG​TGA​AGA​CGC​CAG​TGG​A-3'; cGAS forward: 5′-CCA​ATC​TAA​GAC​GAG​AGC​CGT-3′ and reverse: 5′-GCC​AGG​TCT​CTC​CTT​GAA​AAC​TAT-3'; STING: 5′-CCA​GCC​TGA​TGA​TCC​TTT​GGG-3′ and reverse: 5′-GGC​TAG​GTG​AAG​TGC​TAG​GT-3'; IL1β:forward:5′-GTGTCTTTCCCGTGGACCTT-3′ and reverse: 5′-AAT​GGG​AAC​GTC​ACA​CAC​CA-3'; IL6 forward: 5′-CTT​CTT​GGG​ACT​GAT​GCT​GGT-3′ and reverse: 5′-CTC​TGT​GAA​GTC​TCC​TCT​CCG-3'; TNF-α forward: 5′-CGG​GCA​GGT​CTA​CTT​TGG​AG-3′ and reverse 5′-ACC​CTG​AGC​CAT​AAT​CCC​CT-3'; mtDNA:forward:5′-AAGTTTAACGGCCGCGGTAT-3′ and reverse 5′-AGT​TGG​ACC​CTC​GTT​TAG​CC’3'

### 2.10 Cell Counting Kit 8 cell viability assay

Cells (5 × 104 cells/well) were seeded in a 96-well plate and grown overnight. Next, cells were treated with DMEM, rotenone (0.5 mM), and PA (0.2 mM). Cell viability was detected using a Cell Counting Kit (CCK) eight assay (A311-02, Vazyme, Nanjing, China) (Fan et al., 2024). Briefly, after 24 h, 10 mL of CCK8 solution was added to each well and the incubation continued for another 4 h at 37°C. Absorbance was measured using a multimode microplate reader (Thermo Fisher Scientific Inc., MA, United States) at 450 nm. Cell viability was expressed as a percentage relative to the absorbance of control cells.

### 2.11 Seahorse XF cell Mito Stress test

The mitochondrial functions of cells were measured using a Seahorse XF cell Mito Stress test kit (103,015–100, Agilent Technologies, Santa Clara, CA, United States) and a Seahorse XF24 Extracellular Flux Analyzer (Li et al., 2023). To measure the oxygen consumption rate, mitochondrial complex inhibitors (oligomycin [1 mM], FCCP [1 mM], and rotenone/antimycin A [0.5 mM]) were successively added to the cell culture microplate to measure key parameters of mitochondrial function using the Seahorse XF24 Analyzer. Each sample was assayed based on a minimum of three replicates, and the data were normalized to the protein content in each well.

### 2.12 Statistical analysis

Statistical analysis was performed using GraphPad Prism 5.01 software (GraphPad, Inc., La Jolla, CA, United States). The results are presented as the mean ± S.E. (standard error). All analyses were performed depending on the distribution (parametric or non-parametric) of the data. The Shapiro–Wilk’s test for normality was used for this purpose. If the data followed a normal distribution, they were analyzed using oneway analysis of variance, whereas two-group comparisons were performed using the Student’s t-test. *p*-values <0.05 were considered statistically significant.

## 3 Results

### 3.1 T2DM aggravated motor and cognitive symptoms in PD mouse model

**FIGURE 1:**
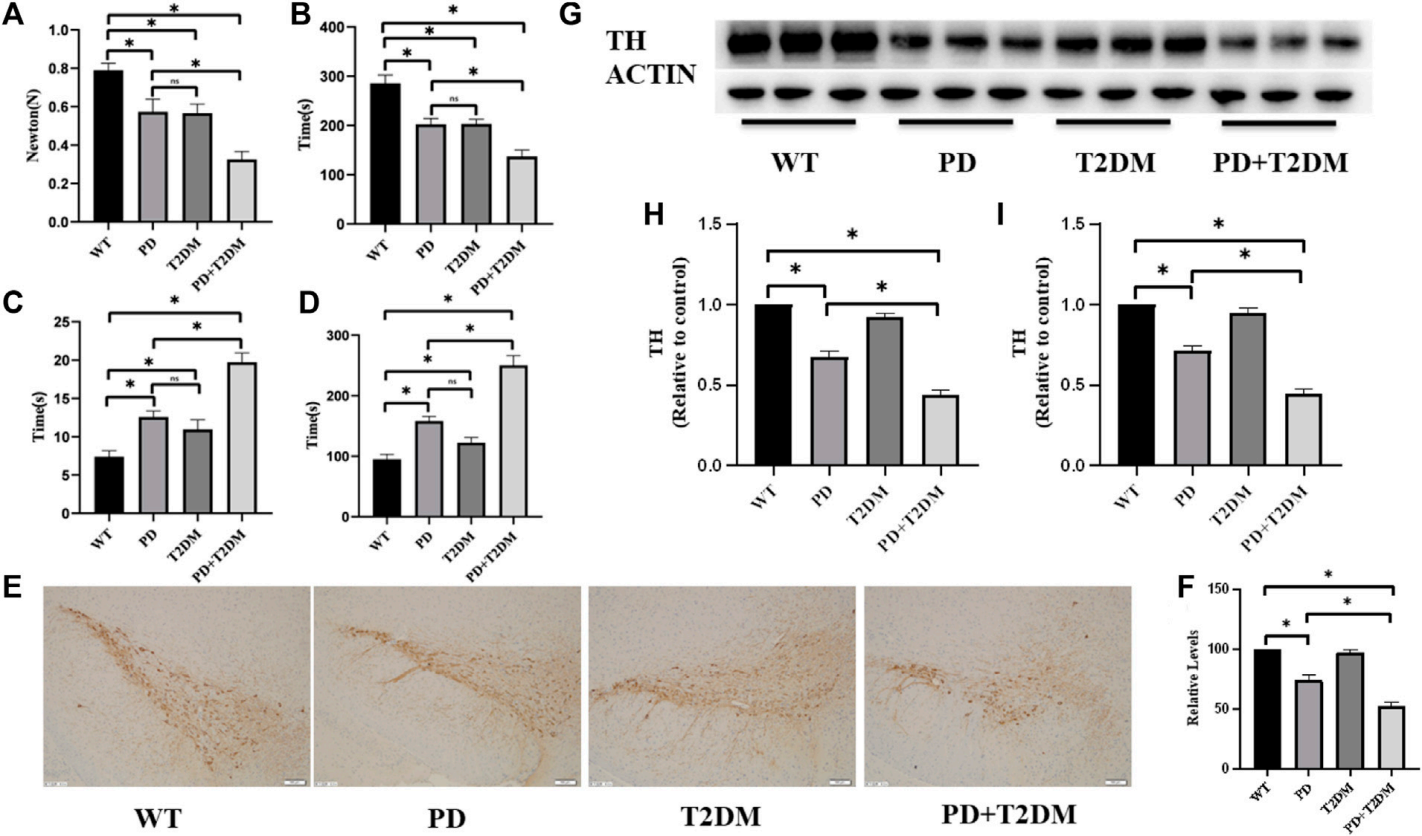
T2DM exacerbates PD motor and cognitive symptoms, leading to progressive loss of dopaminergic neurons. **(A)** Grip strength test results indicate a decrease in forelimb grip strength in PD mouse with T2DM, worse compared to the PD group alone. **(B)** Rotarod test results show a decrease in the duration of time spent on the rod in PD mouse with T2DM, worse than the PD group alone. **(C)** Balance beam test results demonstrate an extended experimental completion time in PD mouse with T2DM, indicating weaker motor abilities compared to the PD group alone. **(D)** Barnes maze test results show an extended exploration time in PD mouse with T2DM, indicating poorer cognitive function compared to the PD group alone. **(E)** Immunohistochemistry results on the substantia nigra region of the four groups of mouse show loss of dopaminergic neurons in the PD group and PD group with T2DM. (The scale is 100 μm) **(F)** Quantitative analysis reveals that the PD group with T2DM has the most severe loss of dopaminergic neurons. **(G, H)** Western blotting results demonstrate lower levels of TH protein in the substantia nigra region of mouse in the PD group and PD group with T2DM compared to the WT group, with the lowest levels observed in the PD group with T2DM. **(I)** qRT-PCR results indicate lower levels of TH mRNA in the substantia nigra region of mouse in the PD group and PD group with T2DM compared to the WT group, with the lowest levels observed in the PD group with T2DM. (*: *p* < 0.05,ns:*p* > 0.05)

To investigate the role of T2DM in the rotenone-induced Parkinson’s disease mouse model, we constructed a mouse model of PD with T2DM and conducted behavioral experiments on mouse to study their phenotypic changes. The pull test revealed that the PD group and PD with T2DM group showed a significant decrease in the distance traveled compared to the WT group and T2DM group, with further reduced grip strength in the PD with T2DM group compared to the PD group (Figure 1A). The rotarod test also showed that the PD group and PD with T2DM group had a shorter maximum duration on the rotarod compared to other groups, with the PD with T2DM group having an even shorter duration than the PD group (Figure 1B). In the balance beam test, it was found that PD with T2DM group took longer time to travel from one end of the balance beam compared to the other three groups (Figure 1C). These experimental results demonstrate that the PD mouse model exhibited motor dysfunction, with T2DM further exacerbating the degree of motor dysfunction in the PD mouse model. Through the Barnes maze experiment, it was found that the PD with T2DM group showed a decrease in accuracy compared to the WT group and PD group. With increasing training sessions and time, the time taken for mouse in the PD with T2DM group to successfully escape gradually decreased, but the time in finding the target box were still higher in the PD with T2DM group compared to the WT group and PD group (Figure 1D). The Barnes maze analysis indicated that T2DM could worsen cognitive dysfunction in the PD mouse model.

### 3.2 T2DM exacerbates the loss of dopaminergic neurons in the substantia nigra region of PD

Tyrosine hydroxylase (TH) is a key enzyme involved in dopamine synthesis and is highly expressed in dopaminergic neurons in the substantia nigra region. The immunohistochemical analysis of TH content in the substantia nigra region of the four groups of mouse showed a reduction in TH protein levels in the PD and PD with T2DM groups, indicating loss of dopaminergic neurons, with the PD with T2DM group showing a more severe damage to dopaminergic neurons than the PD group (Figure 1E–F). Western blotting was used to detect changes in TH protein expression levels, and the results indicated a decrease in TH protein levels in the rotenone-induced PD mouse model, with a more pronounced decrease in the PD with T2DM group (Figure 1G–H). The transcriptional levels of TH mRNA were measured by qRT-PCR, showing the lowest TH mRNA levels in the PD with T2DM group (Figure 1I).

### 3.3 T2DM exacerbates neuroinflammation in the substantia nigra region of PD

**FIGURE 2:**
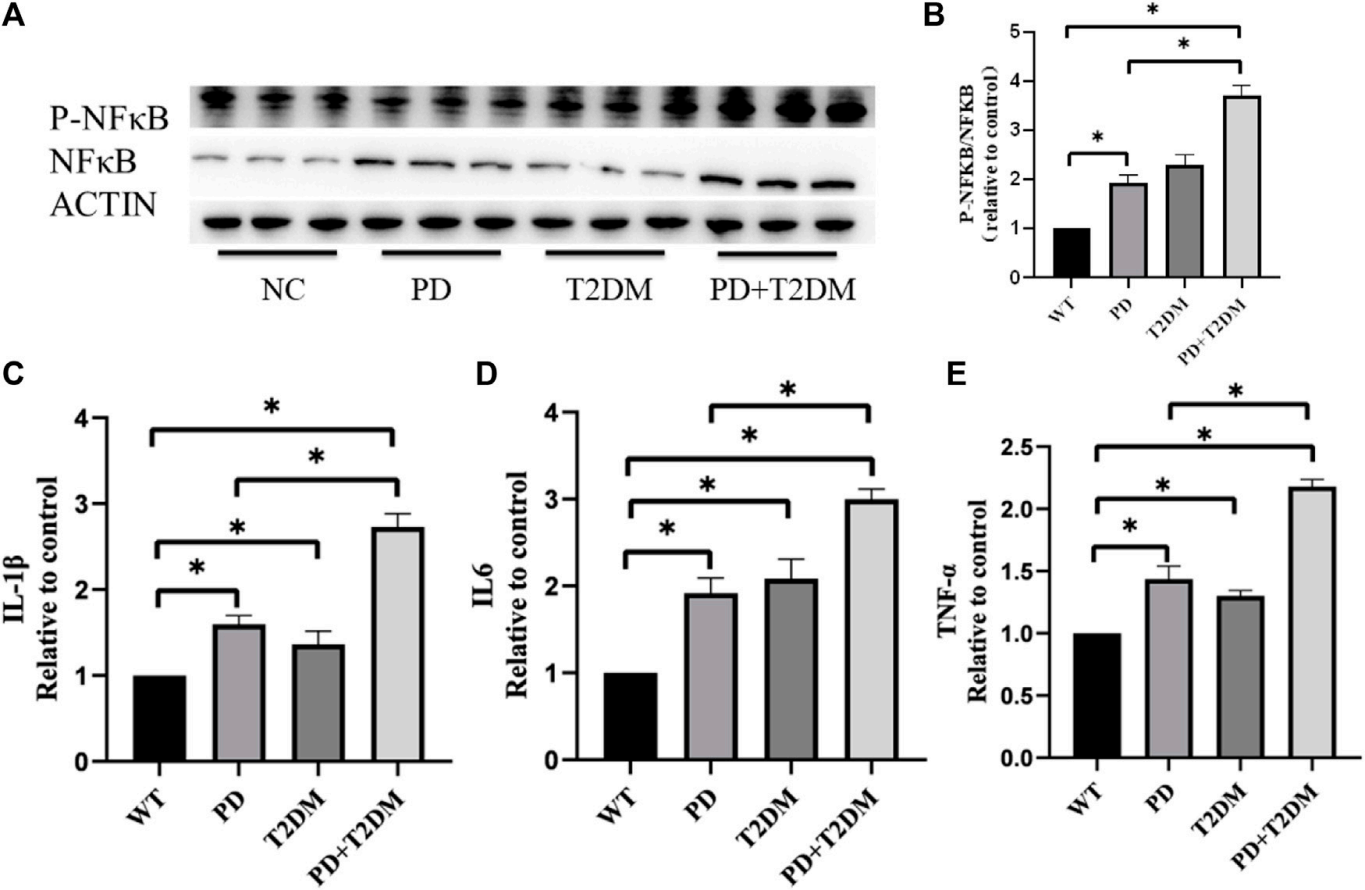
T2DM exacerbates inflammation-related proteins and cytokine expression in the substantia nigra region of PD. **(A, B)** Western blotting results of the four groups of mouse show that the PD with T2DM group has the highest P-NFκB/NFκB protein ratio, followed by T2DM group, then the PD group, and then the WT group. **(C–E)** qRT-PCR results from the substantia nigra region of the four groups of mouse reveal that the PD group with T2DM has the highest levels of TNF-α, IL-1β, and IL-6 cytokines. The PD alone group shows elevated levels of these cytokines compared to the WT group but lower than the PD group with T2DM. (*: *p* < 0.05)

To investigate whether T2DM exacerbates the loss of dopaminergic neurons in PD, the P-NFκB/NFκB levels in the substantia nigra region of the mouse brain were measured by western blotting. The semi-quantitative results showed that the P-NFκB/NFκB ratio was highest in the PD with T2DM group, indicating a potentially severe inflammatory response (Figure 2A,B). qRT-PCR was used to measure the mRNA expression levels of inflammatory factors (IL1-β, IL6, TNF-α) in the brain tissues of the four groups of mouse. The statistical analysis results showed an increase in the expression of inflammatory factors in the PD, T2DM, and PD with T2DM groups, with the PD with T2DM group showing the highest increase in inflammatory factor levels (Figure 2C–E).

### 3.4 T2DM environment activates inflammation in Bv2 cells

**FIGURE 3:**
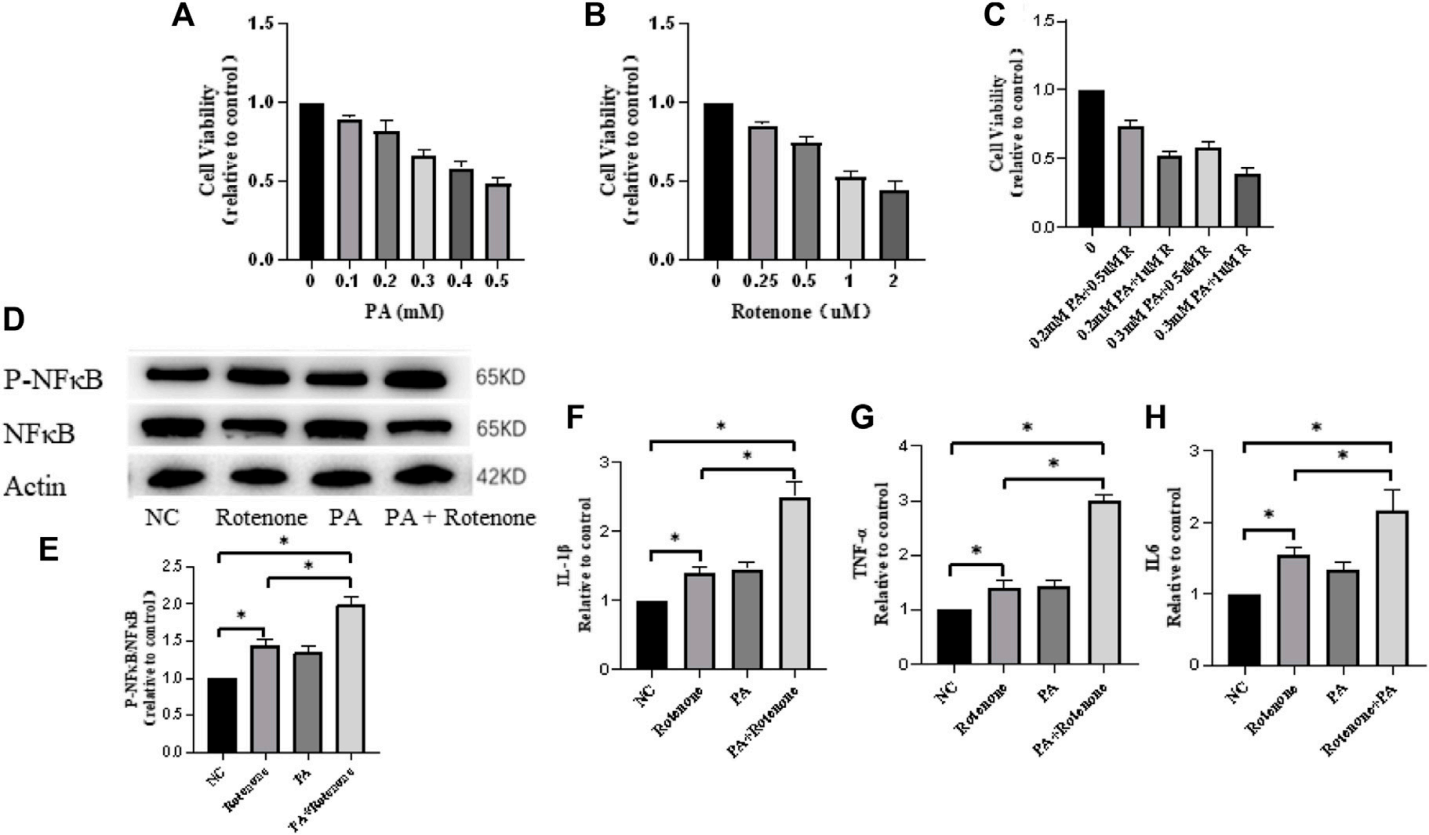
Rotenone and palmitic acid (PA) reduce microglial cell activity and increase expression of inflammation-related proteins and cytokines. **(A–C)** Microglial cell activity decreases with increasing concentrations of Rotenone and PA added to the cells for 48 h **(D, E)** Western blotting results show that the ratio of P-NFκB/NFκB protein is the highest in Rotenone and PA group compared to the three other groups. **(F–H)** qRT-PCR results of the four groups of cells indicate that the Rotenone and PA group has the highest levels of TNF-α, IL-1β, and IL-6 cytokines. The Rotenone group shows elevated levels of these cytokines compared to the NC group but lower than the Rotenone and PA group. (*: *p* < 0.05)

To investigate the role of microglial cells in inflammation, an *in vitro* environment mimicking PD with T2DM in mouse was created using palmitate and rotenone treatments. Bv2 cells were divided into four groups: NC group, rotenone-treated group (PD group), palmitate-treated group (T2DM microenvironment group), and rotenone and PA treated group (T2DM microenvironment and PD group). Cell viability changes were analyzed using a CCK8 assay, showing a decrease in cell viability with increasing palmitate and rotenone concentrations. Considering the impact of cell survival rates on subsequent experiments, a concentration of 0.5uM rotenone was used for the PD group, 0.3 mM palmitate for the T2DM group, and 0.3 mM palmitate +0.5uM rotenone for the PD + T2DM group (Figure 3A–C). Western blotting was used to detect the P-NFκB/NFκB protein ratio in the four groups of cells, showing that the P-NFκB/NFκB expression levels were significantly higher in cells exposed to palmitate and rotenone (Figure 3D–E). qRT-PCR was used to measure the changes in inflammatory factors (IL1-β, IL6, TNF-α) in the four groups of cells, with the results indicating higher levels of inflammatory factors in the rotenone and PA treated group compared to the other three groups (Figures 3F–H). These results demonstrate that T2DM can activate inflammation in BV2 cells, potentially exacerbating PD through this pathway.

### 3.5 T2DM environment impairs mitochondrial function

**FIGURE 4:**
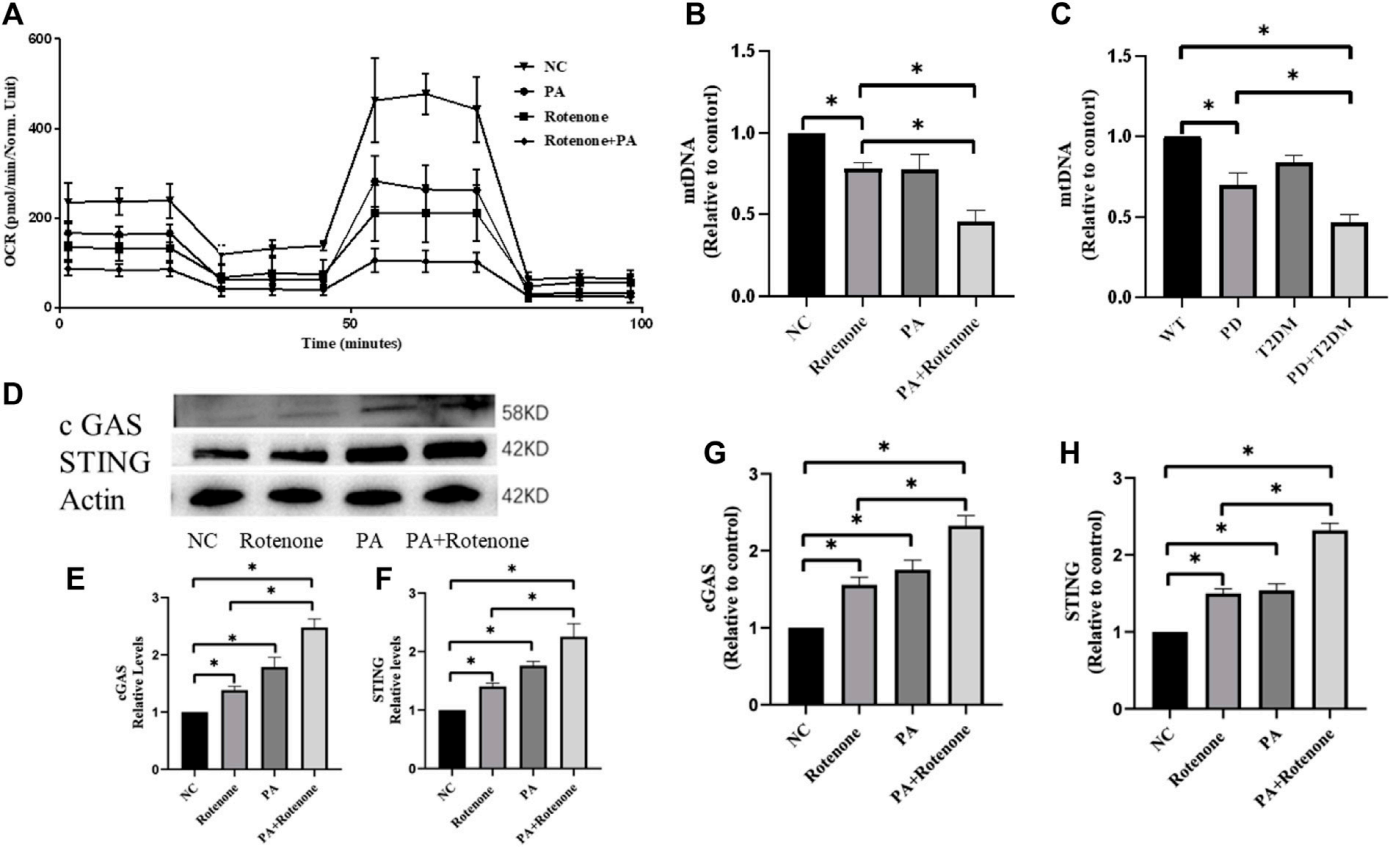
Rotenone plus PA impair mitochondrial function in Bv2 cells and activate the cGAS-STING pathway. **(A)** qRT-PCR results of the four groups of cells show varying degrees of decrease in cellular oxygen pressure, with the Rotenone plus PA group exhibiting the lowest oxygen pressure, suggesting severe mitochondrial damage. **(B)** mtDNA analysis shows a reduction in mitochondrial quantity in all treated groups, with the Rotenone plus PA group showing the most severe decrease. **(C)** mtDNA analysis of the substantia nigra region in the four groups of mouse shows reduced mitochondrial quantity in cells, with the PD group with T2DM exhibiting the most severe mitochondrial damage. **(D–F)** Western blotting results indicate that the Rotenone plus PA group has the highest levels of cGAS and STING proteins, higher than the Rotenone group but lower than the Rotenone plus PA group. **(G, H)** qRT-PCR results show that the Rotenone plus PA group has the highest levels of cGAS and STING mRNA, higher than the Rotenone group but lower than the Rotenone plus PA group. (*: *p* < 0.05)

Considering that both T2DM and PD can affect mitochondrial function and are associated with neuroinflammation, the Seahorse XF Cell Mito Stress Test was used to measure mitochondrial function in the four groups of cells. The results showed different levels of decreased oxygen pressure in the cells of the treatment groups, with the rotenone and PA treatment group showing the greatest decrease in oxygen pressure (Figure 4A). This indicates that mitochondrial function in the treatment groups was compromised, potentially leading to mitochondrial DNA damage. To verify mitochondrial DNA damage, the mtDNA content in the four groups of cells and in the substantia nigra region of the mouse brain was detected by qPCR, showing a decrease in mtDNA content in the treatment group cells and mouse, with the rotenone and PA treated cells having the lowest mtDNA content and the PD with T2DM mouse having the lowest mtDNA content (Figure 4B,C). This suggests that both T2DM and PD can damage mitochondrial function, with T2DM exacerbating the damage to mitochondria in PD.

### 3.6 T2DM environment activates the cGAS-STING pathway in BV2 cells

**FIGURE 5:**
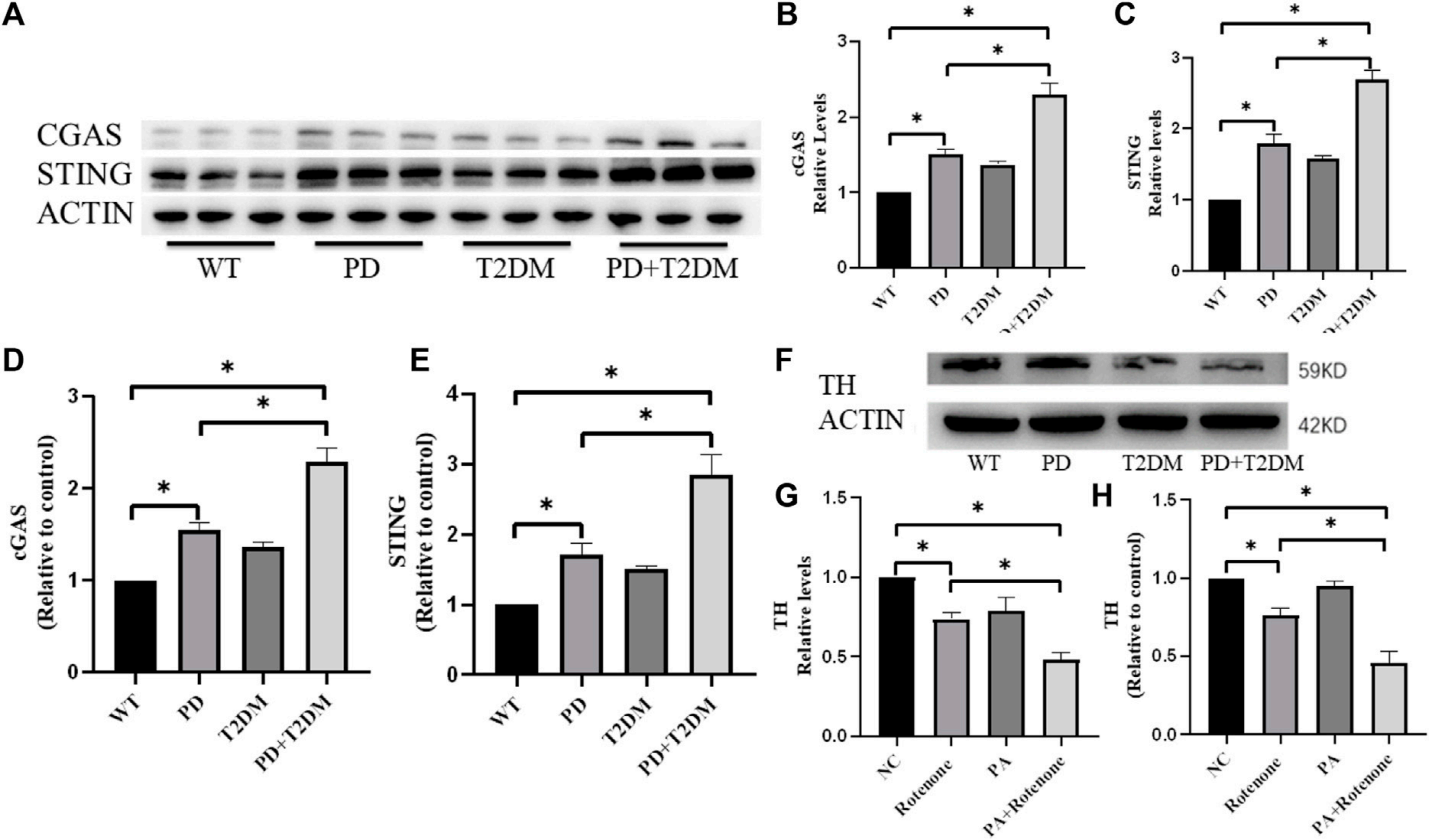
T2DM upregulates cGAS-STING protein expression in a PD mouse model, exacerbating neuronal damage through increased inflammation. **(A–C)** Western blotting results of the substantia nigra region in the four groups of mouse show that the PD group with T2DM has the highest levels of cGAS and STING proteins, followed by the PD group alone, which is higher than the WT group but lower than the PD group with T2DM. **(D, E)** qRT-PCR results of the substantia nigra region in the four groups of mouse show that the PD group with T2DM has the highest levels of cGAS and STING mRNA, followed by the PD group alone, which is higher than the WT group but lower than the PD group with T2DM. **(F–G)** Western blotting results reveal that the supernatant containing inflammatory factors from the Rotenone plus PA group leads to the greatest decrease in TH protein levels in SY5Y cells. (H) qRT-PCR results indicate that the supernatant containing inflammatory factors from the Rotenone plus PA group results in the greatest decrease in TH mRNA levels in SY5Y cells.

To investigate the link between mitochondrial damage and neuroinflammation, western blotting was used to measure the cGAS-STING protein levels in BV2 cells. The results showed that *in vitro* BV2 cells were in an activated state in the cGAS-STING pathway, with higher protein expression levels compared to normal cells, and the rotenone and PA treatment group showing the highest expression levels (Figure 4D–F). qPCR was used to measure the cGAS-STING mRNA levels in the four groups of cells, with the treatment groups showing higher levels than normal cells, and the rotenone and PA treatment group showing the most significant increase (Figure 4G–H). Subsequently, the protein and mRNA levels of cGAS-STING in the substantia nigra brain tissues of the four groups of mouse were verified. Western blotting results indicated higher cGAS-STING protein expression levels in the PD + T2DM mouse model compared to the PD and T2DM groups (Figure 5A–C),with the mRNA level results showing the highest expression of cGAS-STING in the PD + T2DM group (Figure 5D–E). Therefore, it was concluded that T2DM activates the cGAS-STING pathway in BV2 cells, leading to the release of a large number of inflammatory factors.

### 3.7 Inflammatory factors exacerbate damage in SH-SY5Y cells

To determine whether the inflammatory factors released by BV2 cells exacerbate neuronal cell damage, the supernatant containing inflammatory factors from the four groups of BV2 cells was transferred to SH-SY5Y cells modeling PD. Western blotting was used to measure the expression levels of TH protein in the four groups of SH-SY5Y cells, showing a gradual decrease in TH protein levels with increasing inflammatory factor content, with the rotenone and PA treatment group having the lowest TH protein levels (Figure 5F–G). mRNA level testing showed the lowest TH mRNA levels in the rotenone and PA treatment group (Figure 5H). These results demonstrate that the inflammatory factors released by BV2 cells can exacerbate neuronal cell damage.

## 4 Discussion

This study aimed to investigate the relationship between T2DM and PD, and elucidated the mechanism of action of T2DM in the rotenone-induced Parkinson’s disease model from the perspectives of mitochondrial function and neuroinflammation. Rotenone has been increasingly used in PD model construction due to its slow onset pattern resembling PD (Chia 2020; Wrangel et al., 2015). On the other hand, STZ and PA are recognized drugs used to simulate conditions of diabetes both *in vivo* and *in vitro* (Huang et al., 2014; Nath et al., 2017). In this study, we innovatively combined these two chemicals to establish cell and mouse models of PD with T2DM, delving deep into the relationship between the two. We found that T2DM damages the mitochondrial function of the microglial cells, leading to leakage of mt DNA into the cytoplasm. The presence of free mt DNA in the cytoplasm activates the cGAS-STING pathway, and results in releasing a large amount of inflammatory factors. Inflammatory factors produced by microglia aggravate the damage of dopaminergic neurons and promote the progression of PD.

PD is a common neurodegenerative disease with a complex pathogenesis, with previous studies showing a close association between mitochondrial dysfunction and PD (Golpich et al., 2017; Mounsey and Teismann, 2011). Mitochondrial dysfunction in PD has been linked to various factors, including the pathogenic genes PINK/PRKN associated with PD causing mitochondrial dysfunction in neurons (Xu et al., 2020; Wang et al., 2023). Environmental factors such as exposure to organic compounds or pesticides have also been found to disrupt mitochondrial bioenergetics and electron transport chain function, leading to various forms of mitochondrial damage (Jayasundara, 2017; Roubicek and Souza-Pinto, 2017). If cells cannot self-repair mitochondrial dysfunction, it can lead to insufficient cellular energy supply, causing cellular damage and death. T2DM, the most common type of diabetes, has been linked to mitochondrial structural and functional abnormalities, with studies suggesting that mitochondrial damage may be a key factor in addressing the complications caused by T2DM (Patti and Corvera, 2010; Victor et al., 2011). Given the impact of T2DM on the central nervous system, our current research focused on the clinical cognitive impairment of PD with T2DM patients, revealing that this group exhibited cognitive symptoms inferior to those with PD alone. This inspired our hypothesis that T2DM and PD may aggravate mitochondrial damage in the nervous system, affecting the energy supply to dopaminergic neurons and exacerbating PD. Thus, we conducted a series of studies on mitochondrial function, ultimately concluding that T2DM can exacerbate mitochondrial damage in PD (Figures 4A–C).

Mitochondrial damage inevitably affects a series of processes such as mitochondrial biogenesis, fission, and can even lead to mitochondrial rupture with mt DNA leaking into the cytoplasm. Free DNA in the cytoplasm often triggers a series of effects (Duchen, 2004; Apostolova et al., 2011). Studies on PD have found that when nuclear DNA leaks into the cytoplasm, it can activate the cGAS-STING signaling pathway, triggering an autoimmune response and causing neurological inflammation, worsening Parkinson’s disease (Moya et al., 2021; Paul et al., 2021). In aging-related studies, the cGAS-STING pathway plays a crucial role in accelerating neuronal death and promoting aging in humans (Andrade et al., 2022). As a DNA sensor, the cGAS protein can also bind to mt DNA and play a role (Hu et al., 2022). This inspired us to investigate the role of the cGAS-STING pathway in the nervous system and found that T2DM can activate the cGAS-STING pathway in PD (Figures 4D–H; Figures 5A–E).

Neuroinflammation, another major contributing factor to PD, has played a significant role in the occurrence and development of Parkinson’s disease (Lee et al., 2006; PajaresRojo et al., 2020). In PD, damage and death of dopaminergic neurons lead to motor dysfunction. Inflammation may accelerate this process of neuronal damage (Vivekanantham et al., 2015). Research indicates that inflammation-related molecules can trigger oxidative stress responses, disrupt mitochondrial function, and cause neuronal death (Escudero-Lourdes, 2016; Rimessi et al., 2016). Therefore, the exploration of neuroinflammation is crucial in the comprehension of PD pathogenesis. Additionally, T2DM is often with inflammation, which can lead to atherosclerosis and subsequently trigger neurological diseases (Sifuentes-Franco et al., 2017). The specific effects of T2DM on neuroinflammation are still unclear, but our study, through the analysis of inflammatory factors, tentatively suggests that T2DM can activate neuroinflammation in microglial cells (Figures 3D–H), leading to the formulation of a mechanistic pathway involving T2DM-mitochondrial damage-cGAS-STING-neuroinflammation-PD. Future research directions will focus on the protection of mitochondrial function and suppression of neurological inflammation for the treatment of comorbid PD with T2DM.

A recent study published in the New England Journal of Medicine demonstrated the significant effect of the GLP-1 class drug liraglutide in slowing motor impairments in early-stage Parkinson’s disease patients during a 12-month treatment period (Meissner et al., 2024). Another study in 2016 showed that the diabetes drug exenatide improved motor symptoms and cognitive function in PD (Zimmerman et al., 2023). Additionally, basic research has shown that exenatide can improve motor and cognitive impairments in MPTP and 6-OHDA mouse models and in rats with dopamine neuron degeneration induced by lipopolysaccharides (Golpich et al., 2017; Wang et al., 2018). PPARγ agonists such as pioglitazone and rosiglitazone have also been confirmed to exert neuroprotective effects in a range of Parkinson’s disease animal models, significantly improving motor and cognitive impairments in mouse (Lee et al., 2006; Agarwal et al., 2017; Kopp et al., 2022). Other drugs used in the treatment of type 2 diabetes, such as metformin and bromocriptine, have been studied in PD cell lines and mouse models, showing their ability to enhance mitochondrial function and suppress neurological inflammation to provide neuroprotection (Lu et al., 2016; Patil et al., 2014; P et al., 2021). Given the numerous shared mechanisms between T2DM and PD, the therapeutic value of T2DM drugs in the treatment of PD warrants further exploration.

## 5 Conclusion

Our study demonstrated that in the mouse models simulated by rotenone and STZ, as well as in the BV2 cell models simulated by rotenone and PA, the T2DM microenvironment impaired the mitochondrial function of microglial cells, leading to mt DNA leakage, activation of the cGAS-STING pathway, downstream inflammatory reactions, release of a large number of inflammatory factors, and exacerbation of dopaminergic neuron damage in PD.

## Data Availability

The datasets presented in this study can be found in online repositories. The names of the repository/repositories and accession number(s) can be found in the article/supplementary material.

## Abbreviations

PD, Parkinson’s disease; T2DM, type 2 diabetes; TNFα, Tumor necrosis factor-α; IL-1β, Interleukin-1β; IL-6, Interleukin-6; cGAMP, cyclic GMP-AMP; ATP, adenosine triphosphate; GTP, guanosine triphosphate; cGAS, Cyclic GMP-AMP synthase; STING, Stimulator of interferon genes; STZ, Streptozotocin; PA, Palmitate acid; ER, endoplasmic reticulum.
